# Pharmacokinetics and Safety of Omadacycline in Subjects with Impaired Renal Function

**DOI:** 10.1128/AAC.02057-17

**Published:** 2018-01-25

**Authors:** Jolene K. Berg, Evan Tzanis, Lynne Garrity-Ryan, Stephen Bai, Surya Chitra, Amy Manley, Stephen Villano

**Affiliations:** a DaVita Clinical Research, Minneapolis, Minnesota, USA; b Paratek Pharmaceuticals Inc., King of Prussia, Pennsylvania, USA

**Keywords:** dose adjustment, omadacycline, renal impairment

## Abstract

Many antibiotics require dose adjustments in patients with renal impairment and/or in those undergoing hemodialysis. Omadacycline, the first aminomethylcycline antibiotic in late-stage clinical development, displays activity against a broad spectrum of bacterial pathogens, including drug-resistant strains. Data from completed phase 3 studies of omadacycline for the treatment of acute bacterial skin and skin structure infections (ABSSSI) and community-acquired bacterial pneumonia (CABP) showed intravenous (i.v.) to once-daily oral omadacycline to be clinically effective and well tolerated. To determine if the dosing of omadacycline should be adjusted in patients with impaired renal function, a phase 1 study examining the pharmacokinetics (PK) and safety of i.v. omadacycline (100 mg) was conducted in subjects with end-stage renal disease (ESRD) on stable hemodialysis (*n* = 8) and in matched healthy subjects (*n* = 8). i.v. administration of omadacycline produced similar plasma concentration-time profiles in subjects with ESRD and healthy subjects. Further, in subjects with ESRD, similar values of the PK parameters were observed when omadacycline was administered i.v. after or before dialysis. The mean area under the concentration-time curve from time zero extrapolated to infinity in plasma was 10.30 μg · h/ml when omadacycline was administered to ESRD subjects after dialysis, 10.20 μg · h/ml when omadacycline was administered to ESRD subjects before dialysis, and 9.76 μg · h/ml when omadacycline was administered to healthy subjects. The mean maximum observed concentration of omadacycline in plasma in ESRD subjects was 1.88 μg/ml when it was administered after dialysis and 2.33 μg/ml when it was administered before dialysis, and in healthy subjects it was 1.92 μg/ml. The 100-mg i.v. dose of omadacycline was generally safe and well tolerated in both ESRD and healthy subjects. This study demonstrates that no dose adjustment is necessary for omadacycline in patients with impaired renal function or on days when patients are receiving hemodialysis.

## INTRODUCTION

As of 2014, nearly 15% of adults in the United States had some form of chronic kidney disease (CKD), and the prevalence of the most severe form of renal disease, end-stage renal disease (ESRD), continues to rise annually (1). According to the U.S. Renal Data System, there are almost 680,000 cases of ESRD in the United States, and 63% of these patients, about 427,000 individuals, receive hemodialysis (1). Community-acquired infections are of concern in this population; patients with CKD have a nearly 2-fold increase in the risk of pneumonia (2), including community-acquired bacterial pneumonia (CABP). Further, 9.3% of patients who are hospitalized for acute bacterial skin and skin structure infections (ABSSSI) have moderate to severe renal disease (3), and 6% of patients with community-acquired pneumonia present with renal disease as a comorbidity (4). The rate of hospitalization for infections in patients receiving hemodialysis increased 95% between 1993 and 2005 (5).

Omadacycline is the first aminomethylcycline antibiotic in late-stage clinical development. Aminomethylcyclines are semisynthetic antibiotics related to tetracyclines (6, 7; see also the review by Chopra and Roberts [8]). Similar to their tetracycline counterparts, aminomethylcyclines inhibit bacterial protein synthesis. Importantly, however, the 2 main mechanisms of tetracycline resistance, namely, efflux pumps and ribosomal protection, are overcome by modifications present at the C-7 and C-9 positions in the chemical structure of omadacycline (7, 9). Omadacycline has been shown to be active against a variety of bacterial pathogens: Gram-positive aerobes, including methicillin-resistant Staphylococcus aureus (MRSA), penicillin-resistant and multidrug-resistant Streptococcus pneumoniae, and vancomycin-resistant enterococcus (VRE); Gram-negative aerobes; some anaerobes; and atypical bacteria, such as Legionella spp. and Chlamydia spp. (6, 9).

Omadacycline is in clinical development for the treatment of ABSSSI and CABP. In two phase 3 trials, intravenous (i.v.) to once-daily oral omadacycline was found to be effective and noninferior to linezolid (for the treatment of ABSSSI in Omadacycline in Acute Skin and Skin Structure Infections Study 1 [OASIS-1; ClinicalTrials.gov registration no. NCT02378480]) or moxifloxacin (for the treatment of CABP in the Omadacycline for Pneumonia Treatment in the Community trial [OPTIC; ClinicalTrials.gov registration no. NCT02531438]) and to have safety and tolerability similar to those of those two drugs (10, 11). In both studies, the omadacycline dosing regimen was 100 mg i.v. every 12 h for 2 doses followed by 100 mg every 24 h (q24h) for at least 3 days, followed by 300 mg orally q24h. Once-daily oral omadacycline has also shown noninferiority to twice-daily linezolid for the treatment of ABSSSI (OASIS-2, ClinicalTrials.gov registration no. NCT02877927) (12).

While oral omadacycline is eliminated predominantly in the feces, an appreciable portion of the oral dose (14.4% in humans) is eliminated, primarily as unmetabolized omadacycline, in the urine (13). Therefore, understanding the effect of renal impairment on omadacycline is critical for its effective clinical use. This study examined the pharmacokinetics (PK) and safety of omadacycline in patients with ESRD requiring hemodialysis and compared them with those in healthy subjects. We also examined the effect of hemodialysis on the PK of omadacycline by administering omadacycline to the ESRD subjects after or before hemodialysis. The i.v. dose of omadacycline evaluated (100 mg) was the same that was utilized in the phase 3 studies described above. Results from this study provide insight to guide dosing regimens for patients with renal impairment and those receiving hemodialysis.

## RESULTS

### Subject demographics and safety and PK populations.

A total of 44 subjects were screened. Twenty-eight subjects failed the screening and were not enrolled in the study. Sixteen subjects were enrolled, with 8 subjects being assigned to each of the 2 groups (healthy subjects and subjects with ESRD requiring dialysis). All subjects completed the study and were included in both the safety and PK populations. Subject baseline demographic characteristics are summarized in Table 1. The age range across both groups was 43 to 70 years, with the median age being 58.5 years. Overall, 12 subjects (75.0%) were male and 4 subjects (25.0%) were female. A majority (87.5%) of healthy subjects were white, whereas race was more heterogeneous among the subjects with ESRD.

**TABLE 1:** Baseline demographic characteristics^*a*^

Characteristic	Value for:		
	ESRD subjects (*n* = 8)	Healthy subjects (*n* = 8)	Total (*N* = 16)
Median (range) age (yr)	58.5 (43–70)	56.5 (45–67)	58.5 (43–70)
No. (%) of subjects by sex			
Female	2 (25.0)	2 (25.0)	4 (25.0)
Male	6 (75.0)	6 (75.0)	12 (75.0)
No. (%) of subjects by race			
White	4 (50.0)	7 (87.5)	11 (68.8)
Black/African American	3 (37.5)	1 (12.5)	4 (25.0)
Other	1 (12.5)	0	1 (6.3)
No. (%) of subjects by ethnicity			
Hispanic or Latino	1 (12.5)	1 (12.5)	2 (12.5)
Not Hispanic or Latino	7 (87.5)	7 (87.5)	14 (87.5)
Median (range) wt (kg)	87.5 (52.9–129.7)	91.7 (61.2–129.3)	88.9 (52.9–129.7)
Median (range) BMI (kg/m^2^)	28.0 (21.5–39.2)	28.5 (20.9–41.7)	28.5 (20.9–41.7)

a BMI, body mass index; ESRD, end-stage renal disease; *N*, number of subjects; *n*, number of nonmissing observations.

### Pharmacokinetic assessment. (i) Plasma concentrations and values of the PK parameters of omadacycline in ESRD and healthy subjects.

**FIG 1:**
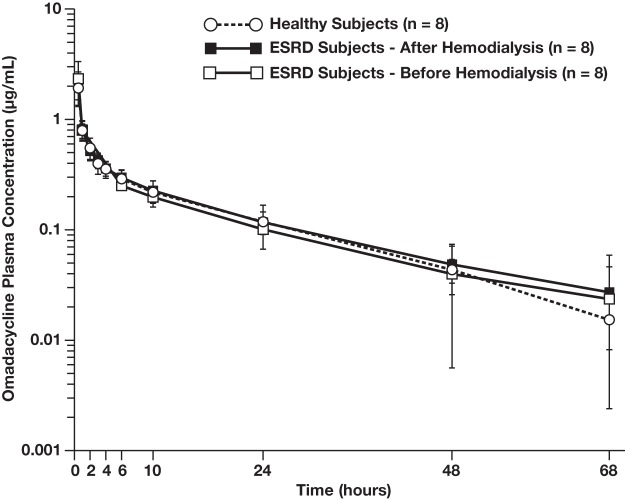
Plasma concentrations of omadacycline in ESRD subjects and healthy subjects (PK population). Subjects were dosed with 100 mg omadacycline i.v. Healthy subjects were dosed once. Subjects with ESRD were dosed after hemodialysis and before hemodialysis, with a 10- to 20-day washout period taking place between doses.

The mean ± standard deviation profiles of the plasma concentrations of omadacycline over time were nearly identical in all cohorts (Fig. 1). Subjects with ESRD displayed a plasma-time concentration profile similar to that of the healthy subjects, irrespective of whether omadacycline was dosed after or before hemodialysis. Further, in all cohorts, omadacycline distributed rapidly in plasma (median time of the maximum observed plasma omadacycline concentration [*T*_max_], ≤1 h) and omadacycline plasma concentrations declined in a biphasic manner.

When omadacycline was dosed after or before hemodialysis in ESRD subjects, the values of all plasma PK parameters in ESRD subjects were similar to those in healthy subjects (Table 2). In terms of total systemic exposure following the i.v. administration of 100 mg omadacycline, the mean area under the concentration-time curve from time zero extrapolated to infinity in plasma (AUC_0–inf_) was 10.30 μg · h/ml and 10.20 μg · h/ml in ESRD subjects when it was dosed after dialysis and before dialysis, respectively, whereas it was 9.76 μg · h/ml in healthy control subjects. Across all 3 cohorts, the mean maximum observed concentration of omadacycline in plasma (*C*_max_) ranged from 1.88 μg/ml to 2.33 μg/ml, the mean terminal elimination half-life in plasma (*t*_1/2_) ranged from 17.1 to 18.9 h, the mean total body clearance (CL) ranged from 10.1 to 10.6 liters/h, and the mean volume of distribution at steady state (*V*_ss_) ranged from 194 to 214 liters.

**TABLE 2:** Summary of omadacycline PK parameters in ESRD and healthy subjects^*a*^

Subject	AUC_0–last_ (μg · h/ml)	AUC_0–inf_ (μg · h/ml)	*C*_max_ (μg/ml)	*T*_max_ (h)	*t*_1/2_ (h)	CL (liters/h)	*V*_ss_ (liters)
ESRD subjects *n* = 8							
Dosing after hemodialysis	9.40 (2.10)	10.30 (2.35)	1.88 (0.74)	0.58 (0.58, 1.08)	18.6 (5.21)	10.1 (2.24)	214 (56.1)
Dosing before hemodialysis	9.25 (1.80)	10.20 (1.99)	2.33 (1.02)	0.59 (0.58, 0.77)	18.9 (6.34)	10.1 (2.05)	194 (69.2)
Healthy subjects *n* = 8	9.08 (1.86)	9.76 (1.77)	1.92 (0.41)	0.58 (0.58, 0.68)	17.1 (2.60)	10.6 (1.99)	204 (47.6)

a The arithmetic mean (standard deviation) is shown for all parameters except *T*_max_, where the median (range [minimum, maximum]) are shown. AUC_0–last_, area under the concentration-time curve from time zero to the last quantifiable concentration in plasma; AUC_0–inf_, area under the concentration-time curve from time zero extrapolated to infinity in plasma; *C*_max_, maximum observed concentration of omadacycline in plasma; CL, total body clearance; ESRD, end-stage renal disease; PK, pharmacokinetics; *t*_1/2_, terminal elimination half-life in plasma; *T*_max_, time of the maximum observed plasma omadacycline concentration; *V*_ss_, volume of distribution at steady state.

When the values of the PK parameters for the ESRD subjects dosed after dialysis were statistically compared to the values of the PK parameters for the healthy subjects, the data showed that renal impairment did not affect overall exposure (area under the concentration-time curve from time zero to the last quantifiable concentration in plasma [AUC_0–last_] and AUC_0–inf_) or CL and had a minimal effect on *C*_max_ (Table 3).

**TABLE 3:** Statistical comparison of PK parameters for ESRD subjects dosed after hemodialysis versus healthy subjects^*a*^

PK parameter	Cohort^*b*^	Geometric mean	Ratio of geometric mean	90% confidence interval for ratio of geometric mean
AUC_0–last_ (μg · h/ml)	ESRD	9.21	1.03	0.86–1.24
	Healthy	8.91		
AUC_0–inf_ (μg · h/ml)	ESRD	10.10	1.05	0.87–1.26
	Healthy	9.61		
*C*_max_ (μg/ml)	ESRD	1.78	0.94	0.72–1.23
	Healthy	1.88		
CL (liters/h)	ESRD	9.91	0.95	0.79–1.14
	Healthy	10.4		

a The ANOVA model included log-transformed PK parameters as the response variables and matched pairs as the random effect, and the fixed effect term was ESRD status. AUC_0–last_, area under the concentration-time curve from time zero to the last quantifiable concentration in plasma; AUC_0–inf_, area under the concentration-time curve from time zero extrapolated to infinity in plasma; *C*_max_, maximum observed concentration of omadacycline in plasma; CL, total body clearance; ESRD, end-stage renal disease; PK, pharmacokinetics.

b ESRD subjects dosed after hemodialysis served as the test cohort for this analysis and healthy subjects served as the reference cohort for this analysis.

### (ii) Omadacycline plasma PK parameters in ESRD subjects dosed after hemodialysis and ESRD subjects dosed before hemodialysis.

The effect of hemodialysis on the PK of omadacycline was evaluated by calculating the relative exposure of the 100-mg i.v. dose administered in ESRD subjects after and before hemodialysis (Table 4). The results indicated that hemodialysis did not have an effect on the overall extent of exposure (AUC_0–last_ and AUC_0–inf_) or CL. While the difference was not statistically significant, *C*_max_ was slightly greater when subjects received omadacycline before hemodialysis than when subjects received omadacycline after hemodialysis or when subjects were healthy.

**TABLE 4:** Statistical comparison of PK parameters for ESRD subjects dosed after versus before hemodialysis^*a*^

PK parameter	Cohort^*b*^	Geometric mean	Ratio of geometric mean	90% confidence interval for ratio of geometric mean
AUC_0–last_ (μg · h/ml)	Before	9.09	0.988	0.948–1.029
	After	9.21		
AUC_0–inf_ (μg · h/ml)	Before	10.00	0.995	0.961–1.030
	After	10.10		
*C*_max_ (μg/ml)	Before	2.18	1.230	0.983–1.536
	After	1.78		
CL (liters/h)	Before	9.95	1.000	0.971–1.040
	After	9.91		

a The ANOVA model included log-transformed PK parameters as the response variable, age and gender as covariates, and subject as the random effect, and the fixed-effect term was period and weight at the baseline. AUC_0–last_, area under the concentration-time curve from time zero to the last quantifiable concentration in plasma; AUC_0–inf_, area under the concentration-time curve from time zero extrapolated to infinity in plasma; *C*_max_, maximum observed concentration of omadacycline in plasma; CL, total body clearance; ESRD, end-stage renal disease; PK, pharmacokinetics.

b ESRD subjects dosed before hemodialysis served as the test cohort and ESRD subjects dosed after dialysis served as the reference cohort for this analysis.

### (iii) Omadacycline urine PK parameters in healthy subjects.

The cumulative amount of omadacycline excreted in the urine of the healthy subjects following dosing with 100 mg of omadacycline i.v. was 27 ± 3.49 mg (mean ± standard deviation). Hence, the fraction of the total omadacycline dose excreted in urine (*Fe_u_*) was 27.0% ± 3.49%. The mean ± standard deviation renal clearance (CL_R_) was 3.06 ± 0.69 liters/h in these subjects.

### (iv) Omadacycline dialysate PK parameters in ESRD subjects dosed before hemodialysis.

During dialysis in ESRD subjects, the mean percentage of omadacycline cleared by hemodialysis following dosing with 100 mg of omadacycline i.v. compared to the total clearance of omadacycline was 47.8%. However, due to the low total systemic clearance (10.1 to 10.6 liters/h across cohorts) and the large volume of distribution (194 to 214 liters across cohorts) of omadacycline, the actual percentage of the omadacycline dose recovered in the dialysate during dialysis was only 7.89% (7.89 mg).

### Safety.

Overall, 5 of 16 subjects enrolled in the study (31.3%) experienced at least 1 treatment-emergent adverse event (TEAE) during the study (Table 5). There were no serious adverse events (SAEs) or deaths reported. Further, no subjects withdrew from the study due to an adverse event (AE), and no AEs resulted in study drug discontinuation or interruption.

**TABLE 5:** Overall summary of TEAE^*a*^

Type of TEAE observed	No. (%) of subjects			
	ESRD subjects (*n* = 8)		Healthy subjects (*n* = 8)	Total (*N* = 16)
	Dosing after hemodialysis	Dosing before hemodialysis		
Any TEAE	3 (37.5)	2 (25.0)	1 (12.5)	5 (31.3)
Upper RTI	2 (25.0)	0	0	2 (12.5)
Viral upper RTI	1 (12.5)	0	0	1 (6.3)
Dizziness	1 (12.5)	0	0	1 (6.3)
Headache	0	1 (12.5)	0	1 (6.3)
Infusion site erythema	0	0	1 (12.5)	1 (6.3)
Bronchospasm	0	1 (12.5)	0	1 (6.3)
Papular rash	1 (12.5)	0	0	1 (6.3)

a ESRD, end-stage renal disease; *N*, number of subjects in group; *n*, number of subjects; RTI, respiratory tract infection; TEAE, treatment-emergent adverse event.

Three TEAEs involved respiratory tract infections, and all 3 were considered by the investigator to be not related to the study treatment. The investigator indicated that all of these respiratory tract infections occurred during the cold season, resolved spontaneously, and were likely to be of viral origin. The event of bronchospasm occurred in an ESRD subject with a history of asthma. Only 1 subject experienced any TEAE considered to be drug related. This subject had ESRD and experienced 2 drug-related TEAEs (dizziness and papular rash) in association with the dosing of omadacycline after dialysis. All subjects experiencing an AE recovered, and all AEs resolved.

There was a transient increase in heart rate that typically peaked within 2 h postdosing in all groups. The largest median increase in heart rate at any measured time point within 24 h (9.5 beats per minute) was observed in healthy subjects. There were no clinically relevant adverse trends in blood pressure, other than a transient increase observed at about the time of initiation of hemodialysis in the ESRD subjects when they were dosed before hemodialysis. No changes in vital sign parameters were reported as AEs. There were no clinically relevant trends in the hematology, clinical chemistry, or 12-lead electrocardiogram (ECG) results.

## DISCUSSION

A comparison of the PK data for omadacycline for ESRD subjects versus healthy subjects in the present study suggests that dose adjustment is not necessary in patients with any degree of renal impairment, including those receiving hemodialysis. Intravenous administration of omadacycline produced similar plasma concentration-time profiles and values of the PK parameters in ESRD subjects on hemodialysis and in matched healthy subjects. Even though 27% of the dose was eliminated in the urine of healthy subjects, the overall clearance and volume of distribution were similar in ESRD subjects and healthy subjects. The pharmacokinetic findings (including clearance, volume of distribution, and the fraction of the dose excreted unchanged in urine) from the healthy subject cohort in the present study were similar to those reported previously (13–15). When subjects with ESRD received i.v. omadacycline after or before hemodialysis, no significant differences were observed. There was no effect on the overall extent of exposure (AUC_0–last_ and AUC_0–inf_), CL, *V*_ss_, or *t*_1/2_. The effect on *C*_max_ was considered small and not clinically relevant. Omadacycline was generally safe in healthy subjects, as has been previously observed (13, 16), and in subjects with ESRD. Only 1 subject presented with mild TEAEs related to omadacycline, and no SAE was reported.

The present findings with omadacycline are significant, considering that the PK of many antibiotics is altered in patients with renal impairment or undergoing hemodialysis. Appropriate antibiotic dosing is an appreciated clinical challenge in these patient populations (17). Administration of drugs with significant renal clearance to patients with renal impairment, without dosage adjustment, may lead to overdosing, wherein the decreased drug clearance leads to an increase in the overall exposure and a potential increase in the incidence of adverse events. In turn, this can potentially lead to increased mortality and a greater burden on the health care system in the form of increased hospitalizations, an increased length of hospital stay, and increased clinical investigations (18). Conversely, drugs that are hydrophilic and usually subject to renal clearance may be filtered out during hemodialysis, thus decreasing the overall exposure and increasing the opportunity for underdosing. Underdosing may lead to the decreased efficacy of treatment and allow the possibility of the development of antibiotic resistance. To avoid this, patients frequently need supplemental dosing during or following hemodialysis or adjustment of the time of antibiotic administration relative to the time of hemodialysis treatment. Thus, modifications in dosing regimens for the renally impaired patient population and patients undergoing hemodialysis are often required to maintain the appropriate therapeutic concentrations of drug in serum, maximizing the therapeutic potential of the drug while minimizing side effects and adverse events.

Many antibiotics commonly used for the treatment of community-acquired bacterial infections, such as ABSSSI and CABP, require dose adjustments in patients with renal impairment. In patients with any level of renal dysfunction (creatinine clearance [CL_CR_], <90 ml/min), vancomycin, very commonly considered a “gold standard” drug for the treatment of ABSSSI involving MRSA, requires a dose adjustment (19). Telavancin, ceftaroline, levofloxacin, and cefpodoxime all require dosing modifications in patients with moderate renal impairment (CL_CR_, <50 ml/min) (20–23). In patients with the most severe renal impairment (CL_CR_, <30 ml/min), daptomycin, dalbavancin, trimethoprim-sulfamethoxazole, clarithromycin, and amoxicillin require dose adjustments (24–28). Additionally, some antibiotics, such as the beta-lactams, including ceftaroline and cefpodoxime, are filtered out during hemodialysis (21, 23). Thus, efficient dosing of these drugs is problematic in this setting.

In contrast, data from the present study demonstrate that changes to the dosing regimen of omadacycline are not necessary in patients with ESRD on hemodialysis, and therefore, dose adjustment is not necessary for individuals with any degree of renal impairment. Taken together with the findings of previous studies, which report that omadacycline does not require a dose adjustment on the basis of age, sex, or level of hepatic function (29, 30), along with its availability as a once-daily oral administration, the latest findings presented here further support omadacycline as a safe antibiotic that is convenient to dose in diverse patient populations.

## MATERIALS AND METHODS

### Study design.

This was a phase 1, open-label, single-dose, two-period, parallel-group study. The primary objective of the study was to compare the PK of omadacycline in adult subjects with ESRD on hemodialysis to those in matched healthy adult subjects. Secondary objectives were to evaluate the safety and tolerability of single i.v. doses of omadacycline administered to subjects with ESRD, to determine the proportion of omadacycline removed by hemodialysis, and to determine the urine concentration of omadacycline after i.v. administration in healthy subjects. Subjects were assigned to a treatment group depending on renal function status. The IntegReview (Austin, TX, USA) Institutional Review Board (IRB) reviewed and approved this study and its conduct at the clinical site. The IRB was appropriately constituted in accordance with the International Conference on Harmonization (ICH) guideline for good clinical practice (GCP) and local requirements, as applicable. All participants were informed verbally and in writing of the objectives, procedures, and risks of study participation. Participants voluntarily provided written, informed consent prior to undergoing any study-related procedures. The study was conducted from November 2015 (when the first subject was enrolled) to May 2016 (when the last subject completed the study).

### (i) Subject selection.

*(a) Key inclusion criteria*. Subjects who fulfilled the following criteria were eligible for inclusion in the study: male or female, an age 18 years of age or older, and a body weight of ≥50 kg. Healthy subjects were required to have an adequate creatinine clearance, as calculated by the Cockcroft-Gault formula, of ≥90 ml/min and to be in good general health, as determined by the medical history, a physical examination, their vital signs, and the results from an ECG and laboratory tests. Subjects with ESRD were required to be on a stable hemodialysis program (defined as a urea clearance by time divided by the urea volume [*Kt*/*V*] value above 1.2 within the past 4 weeks without a significant change in the past 3 months) and to have acceptable vital signs and a stable ECG. Further, there should have been no evidence of hepatic decompensation, including alanine aminotransferase (ALT) and aspartate aminotransferase (AST) levels ≤3 times the upper limit of normal (ULN) and a bilirubin level ≤1.5 times the ULN. Healthy adult subjects were matched to adult subjects with ESRD on the basis of sex, age (±5 years), and weight (±10 kg).

*(b) Key exclusion criteria*. Healthy subjects were excluded if there was a history of clinically significant cardiac rhythm abnormalities, lab tests indicating liver disease or injury, a history or the presence of impaired renal function, signs of urinary obstruction/difficulty voiding at screening, a recent or recurrent history of acute or chronic bronchospastic disease, or any surgical or medical condition which the investigator believed may have altered the absorption, distribution, metabolism, or excretion of drugs. Subjects with ESRD were excluded from the study if they had a history of congestive heart failure, significant coronary artery disease or unstable angina within the 6 months prior to the screening, an emergency room visit or hospitalization for chest pain or shortness of breath within 2 months of the screening, or a history or evidence of autonomic dysfunction not related to hemodialysis within the preceding 1 year. Key exclusion criteria for both groups included the use of other investigational drugs within 5 half-lives or within 30 days prior to the first dose of study drug, a history of hypersensitivity or allergic reaction to any tetracycline, a history of malignancy of any organ system (other than localized basal cell carcinoma of the skin) within the last 5 years, known HIV infection, and a history of drug or alcohol abuse within 12 months.

### (ii) Treatments.

Each subject with ESRD participated in the study for approximately 65 days. This included a screening period (not exceeding 28 days), a 1-day baseline period, a 4-day treatment period (the first treatment period), and a washout period of 10 to 20 days, followed by a second 1-day baseline period and 4-day treatment period (the second treatment period). After the second treatment period, there was a study completion evaluation which occurred approximately 1 week (±3 days) after the last dose of study drug. In the first treatment period, all ESRD subjects received a single dose of 100 mg omadacycline via i.v. infusion over approximately 30 min on day 1 at 0 to 2 h after dialysis. This dose was given in association with either the Friday or Saturday dialysis session to ensure a 72-h gap from the start of the omadacycline infusion before the next dialysis session. After the washout period, ESRD subjects received a second dose of omadacycline (the second treatment period) approximately 60 to 90 min before dialysis. In both treatment periods, blood samples were collected at specified times through 68 h postdose. During the second treatment period, dialysate samples were collected at specified times through 4 h postdose.

Each healthy subject participated in the study for approximately 40 days. This included a screening period (not exceeding 28 days), a 1-day baseline period, a 4-day treatment period, and a study completion evaluation which occurred approximately 1 week (±3 days) after the last dose of study drug. The healthy subjects received a single i.v. dose of 100 mg omadacycline on day 1. Healthy subjects did not receive a second dose of omadacycline. Blood and urine were collected at specified time points through 72 h postdose. All subjects (ESRD and healthy subjects) were confined to the clinical research unit during the baseline and treatment periods.

### Study assessments. (i) Pharmacokinetic analysis.

The PK population consisted of all subjects who received the intended dose of omadacycline for a given treatment period and for whom the values of the PK parameters could be calculated. This population was used for the PK concentration and PK parameter data summaries. Plasma, urine, and dialysate samples were analyzed for determination of the omadacycline concentration using a validated liquid chromatography-mass spectrometry/mass spectrometry (LC-MS/MS) analytical method at Q2 Solutions, formerly Quintiles BioSciences (Ithaca, NY). The values of the following PK parameters for this population were calculated using WinNonlin PK software (version 5.0 or later): AUC_0–last_, AUC_0–inf_, *C*_max_, *T*_max_, *t*_1/2_, CL, and *V*_ss_.

### (ii) Statistical analysis of pharmacokinetic data.

For estimation of the values of the PK parameters for the 2 groups, statistical analysis of the values of the PK parameters was carried out by using an analysis of variance (ANOVA) model on the log-transformed values of the PK parameters AUC_0–last_, AUC_0–inf_, *C*_max_, and CL as the response variables with the fixed-effect term ESRD status (matched healthy subjects and subjects on hemodialysis [with dosing after dialysis]) and matched pair as the random effect. The estimated mean difference and their associated 90% confidence intervals (CI) were constructed for the differences between ESRD subjects on hemodialysis (dosing after dialysis) and matched healthy subjects. The estimated mean difference and 90% CI were then back-transformed to give estimates and the 90% CI for the ratio of the parameters in both categories, ESRD subjects versus matched healthy subjects.

In addition, to evaluate the effect of dialysis on omadacycline, the log-transformed values of the PK parameters (AUC_0–last_, AUC_0–inf_, *C*_max_, and CL) obtained with dosing before hemodialysis (test) versus dosing after hemodialysis (reference) in ESRD subjects were evaluated using an ANOVA model with period as the factor, body weight at the baseline, age, and gender as covariates, and subject as the random effect. The 2-sided 90% CI for the estimated ratio of the test value versus the reference value was calculated for all PK parameters (AUC_0–last_, AUC_0–inf_, *C*_max_, and CL). The ratio of the geometric means and their CI were obtained by back-transforming the estimated mean difference and its corresponding CI.

### (iii) Safety assessment.

The safety and tolerability of omadacycline were assessed in terms of adverse events (AEs), serious adverse events (SAEs), vital signs, and the findings from a 12-lead electrocardiogram (ECG), a physical examination, and standard clinical laboratory safety tests (hematology and blood chemistry tests, coagulation, and urinalysis [healthy subjects]). The safety population consisted of all enrolled subjects who received any dose of omadacycline. All AEs were listed individually for each subject and summarized by system organ class (SOC) and preferred term (PT) assigned to the AEs and were coded using the *Medical Dictionary for Regulatory Activities* (MedDRA), version 18.0 or later.

## References

[1] U.S. Renal Data System. 2016 2016 USRDS annual data report: epidemiology of kidney disease in the United States. National Institutes of Health, Bethesda, MD.

[2] Chou CY, Wang SM, Liang CC, Chang CT, Liu JH, Wang IK, Hsiao LC, Muo CH, Huang CC, Wang RY 2014 Risk of pneumonia among patients with chronic kidney disease in outpatient and inpatient settings: a nationwide population-based study. Medicine (Baltimore) 93:e174. doi:10.1097/MD.0000000000000174. 25501062 PMC4602797

[3] Kaye KS, Patel DA, Stephens JM, Khachatryan A, Patel A, Johnson K 2015 Rising United States hospital admissions for acute bacterial skin and skin structure infections: recent trends and economic impact. PLoS One 10:e0143276. doi:10.1371/journal.pone.0143276. 26599005 PMC4657980

[4] Ruiz M, Ewig S, Marcos MA, Martinez JA, Arancibia F, Mensa J, Torres A 1999 Etiology of community-acquired pneumonia: impact of age, comorbidity, and severity. Am J Respir Crit Care Med 160:397–405. doi:10.1164/ajrccm.160.2.9808045. 10430704

[5] Collins AJ, Foley RN, Gilbertson DT, Chen SC 2009 The state of chronic kidney disease, ESRD, and morbidity and mortality in the first year of dialysis. Clin J Am Soc Nephrol 4(Suppl 1):S5–S11. doi:10.2215/CJN.05980809. 19996006

[6] Villano S, Steenbergen J, Loh E 2016 Omadacycline: development of a novel aminomethylcycline antibiotic for treating drug-resistant bacterial infections. Future Microbiol 11:1421–1434. doi:10.2217/fmb-2016-0100. 27539442

[7] Honeyman L, Ismail M, Nelson ML, Bhatia B, Bowser TE, Chen J, Mechiche R, Ohemeng K, Verma AK, Cannon EP, Macone A, Tanaka SK, Levy S 2015 Structure-activity relationship of the aminomethylcyclines and the discovery of omadacycline. Antimicrob Agents Chemother 59:7044–7053. doi:10.1128/AAC.01536-15. 26349824 PMC4604364

[8] Chopra I, Roberts M 2001 Tetracycline antibiotics: mode of action, applications, molecular biology, and epidemiology of bacterial resistance. Microbiol Mol Biol Rev 65:232–260. doi:10.1128/MMBR.65.2.232-260.2001. 11381101 PMC99026

[9] Draper MP, Weir S, Macone A, Donatelli J, Trieber CA, Tanaka SK, Levy SB 2014 Mechanism of action of the novel aminomethylcycline antibiotic omadacycline. Antimicrob Agents Chemother 58:1279–1283. doi:10.1128/AAC.01066-13. 24041885 PMC3957880

[10] O'Riordan WA, Green S, Overcash JS, Pulijiz I, Metallidis S, Gardovskis J, Garrity-Ryan L, Das A, Tzanis E, Eckburg P, Manley A, Villano S, Loh E 2017 A phase 3 randomized, double-blind, multi-center study to compare the safety and efficacy of oral and i.v. omadacycline to linezolid for treating adult subjects with ABSSSI (the OASIS study), abstr 630. Abstr 27th Eur Congr Clin Microbiol Infect Dis (ECCMID), Vienna, Austria.

[11] Globe Newswire. 3 4 2017 Paratek announces positive phase 3 study of omadacycline in community-acquired bacterial pneumonia. Press release. Globe Newswire, Boston, MA.

[12] Globe Newswire. 17 7 2017 Paratek announces phase 3 study of oral-only dosing of omadacycline met all primary and secondary FDA and EMA efficacy endpoints in acute bacterial skin infections. Press release. Globe Newswire, Boston, MA.

[13] Flarakos J, Du Y, Gu H, Wang L, Einolf HJ, Chun DY, Zhu B, Alexander N, Natrillo A, Hanna I, Ting L, Zhou W, Dole K, Sun H, Kovacs SJ, Stein DS, Tanaka SK, Villano S, Mangold JB 2017 Clinical disposition, metabolism and in vitro drug-drug interaction properties of omadacycline. Xenobiotica 47:682–696. doi:10.1080/00498254.2016.1213465. 27499331

[14] Overcash JS, Bhiwandi P, Tzanis E, Garrity-Ryan L, Steenbergen J, Bai S, Chitra S, Manley A, Villano S 2017 Pharmacokinetics and safety of omadacycline in patients with uncomplicated urinary tract infections, poster Sunday 200. Abstr Microbe 2017, New Orleans, LA. American Society for Microbiology, Washington, DC.

[15] Tanaka SK, Steenbergen J, Villano S 2016 Discovery, pharmacology, and clinical profile of omadacycline, a novel aminomethylcycline antibiotic. Bioorg Med Chem 24:6409–6419. doi:10.1016/j.bmc.2016.07.029. 27469981

[16] Sun H, Ting L, Machineni S, Praestgaard J, Kuemmell A, Stein DS, Sunkara G, Kovacs SJ, Villano S, Tanaka SK 2016 Randomized, open-label study of the pharmacokinetics and safety of oral and intravenous administration of omadacycline to healthy subjects. Antimicrob Agents Chemother 60:7431–7435. doi:10.1128/AAC.00774-16. 27736760 PMC5119026

[17] Eyler RF, Mueller BA 2010 Antibiotic pharmacokinetic and pharmacodynamic considerations in patients with kidney disease. Adv Chronic Kidney Dis 17:392–403. doi:10.1053/j.ackd.2010.05.007. 20727509

[18] Sultana J, Cutroneo P, Trifiro G 2013 Clinical and economic burden of adverse drug reactions. J Pharmacol Pharmacother 4:S73–S77. doi:10.4103/0976-500X.120957. 24347988 PMC3853675

[19] Pfizer Inc. 2013 Vancomycin package insert. Pfizer Inc, New York, NY.

[20] Theravance Biopharma US, Inc. 2016 Vibativ (telavancin) package insert. Theravance Biopharma US, Inc, South San Francisco, CA.

[21] Forest Pharmaceuticals, Inc. 2016 Teflaro (ceftaroline fosamil) package insert. Forest Pharmaceuticals, Inc, Parsippany, NJ.

[22] Janssen Pharmaceuticals, Inc. 2017 Levaquin (levofloxacin) package insert. Janssen Pharmaceuticals, Inc, Titusville, NJ.

[23] Pharmacia & Upjohn Company. 2013 Vantin (cefpodoxime proxetil) package insert. Pharmacia & Upjohn Company, New York, NY.

[24] Merck Sharp & Dohme Corp. 2017 Cubicin (daptomycin for injection) package insert. Merck Sharp & Dohme Corp, Whitehouse Station, NJ.

[25] Durata Therapeutics U.S. Limited. 2016 Dalvance (dalbavancin) package insert. Durata Therapeutics U.S. Limited, Parsippany, NJ.

[26] Caraco Pharmaceutical Laboratories, Ltd. 2016 Bactrim (sulfamethoxazole and trimethoprim DS) package insert. Caraco Pharmaceutical Laboratories, Ltd, Detroit, MI.

[27] AbbVie Inc. 2016 Biaxin (clarithromycin) package insert. AbbVie Inc, North Chicago, IL.

[28] Dr. Reddy's Laboratories Tennessee LLC. 2015 Amoxil (amoxicillin) package insert. Dr. Reddy's Laboratories Tennessee LLC, Bristol, TN.

[29] Tanaka SK, Tzanis E, Villano S 2016 Effect of age and gender on the pharmacokinetics of the oral and i.v. omadacycline, a new class of aminomethylcyclines, poster P1318. Abstr 26th Eur Congr Clin Microbiol Infect Dis, Amsterdam, The Netherlands.

[30] Ting L, Kovacs SJ, Praestgaard J, Maietta R, Stein DS, Sunkara G, Draper MP, Sun H 2012 An open-label study of the pharmacokinetics and safety of single i.v. and oral doses of omadacycline in subjects with mild, moderate, and severe hepatic impairment, poster A-1282. Abstr 52nd Intersci Conf Antimicrob Agents Chemother, San Francisco, CA. American Society for Microbiology, Washington, DC.

